# Multimodal Prediction and Intervention of Internet Gaming Disorder

**DOI:** 10.2174/011570159X390484250417063434

**Published:** 2025-04-24

**Authors:** Qinghua He, Hanlin Cheng

**Affiliations:** 1 Faculty of Psychology, Southwest University, Chongqing, China

## 1 INTRODUCTION

Internet Gaming Disorder (IGD) is a notable psychological concern impacting adolescents globally, with a particular prevalence in China [1]. The rapid expansion of the internet and video games has amplified concerns about the mental and behavioral well-being of youth in the digital era. Research by Gou *et al*. [2] suggested that IGD affects around 10% of Chinese adolescents, with varying prevalence rates, notably higher in urban areas. The issue not only poses public health challenges but also social implications, influencing social integration, academic performance, and emotional well-being. Associated problems, such as low self-esteem, isolation, aggression, sleep disorders, and academic decline, have raised widespread concerns [3]. Despite governmental initiatives, IGD remains a significant threat to the mental health of young individuals.

As a prevalent psychological and behavioral phenomenon, IGD involves a complex interaction of cognitive, neurological, genetic, and environmental factors. It is not only characterized by excessive dependence on gaming but also closely related to individual cognitive control, emotional regulation, and social interaction mechanisms [4]. This complexity leads to diverse and individualized causes of IGD. In recent years, researchers have used various methods and technologies, including surveys, cognitive tasks, neuroimaging (*e.g*., EEG, fMRI), and brain stimulations (*e.g*., rTMS, tDCS), to significantly advance our understanding of the cognitive mechanisms and neural foundations of IGD. However, most studies are limited to single-level or modal analysis, impeding a comprehensive exploration of IGD complexities. Particularly, cross-sectional studies, while revealing correlations at specific time points, lack predictive information necessary for personalized intervention strategies. Relying on single methods at isolated time points obstructs a holistic comprehension of long-term changes in IGD individuals, hampering timely and accurate predictions for early intervention.

The emergence of multimodal data research in recent years has opened up new avenues for addressing these issues. Multimodal data involves integrating various sources of information, such as behavioral data, neuroimaging, psychological measurements, and physiological signals, to conduct a more thorough and systematic analysis of individual behavior and related physiological responses [5, 6]. For example, the use of functional connectome gradient techniques, which combine brain data with behavioral data, has allowed researchers to pinpoint specific neurobiological markers in individuals at heightened risk of developing IGD [7]. Current evidence identifies three distinct subtypes of IGD: (1) the executive dysfunction subtype, characterized by hypo-connectivity in the prefrontal control network and impaired decision-making; (2) the reward-sensitive subtype, exhibiting hyperactivation in the limbic system and persistent gaming cravings; and (3) the mixed subtype, which combines both neural patterns and presents the most severe symptoms. This tripartite classification system elucidates the heterogeneity of IGD, providing valuable insights for personalized intervention strategies, prognostic assessments, and early risk identification. These findings underscore the potential of combining neuroimaging data with behavioral observations to enhance understanding of the neurocognitive elements contributing to IGD.

Big data analytics provides a new technical approach for predicting IGD. By combining various digital footprints, such as gaming logs, device interactions, and social media activities, researchers can develop more precise risk assessment models. Machine learning algorithms provide notable advantages in analyzing such intricate behavioral data, facilitating the detection of behavioral patterns that traditional measurement approaches may miss [8]. It is important to highlight that the accuracy of these prediction models heavily relies on the standardization of data collection and the representativeness of the sample.

Furthermore, the introduction of artificial intelligence (AI) algorithms has provided new possibilities for processing and analyzing multimodal data. AI technology can deeply mine these vast data sets, utilizing pattern recognition and data integration for early identification and accurate prediction [8]. This data integration helps to identify high-risk adolescents and provides a basis for personalized interventions. The advancement of multimodal technologies presents significant potential for enhancing the early prediction and intervention of IGD. Progress in big data analytics, AI, and machine learning is facilitating the integration of diverse multimodal data types [8], including genetic data, environmental factors, and social influences. This integration can aid in identifying individuals at higher risk of developing IGD. The advancement of precision medicine is leading to more personalized intervention strategies that consider various individual characteristics, including behavioral manifestations, brain structure, and function. Non-invasive brain stimulation techniques, such as transcranial direct current stimulation (tDCS), transcranial alternating current stimulation (tACS), temporal interference stimulation, and repetitive transcranial magnetic stimulation (rTMS), show promise in improving executive function, regulating neural oscillations, targeting deep nuclei precisely, and modulating reward circuit hyperactivity [9-11]. These techniques can complement cognitive behavioral therapy by combining pharmacological treatments, real-time neurofeedback, and individualized brain-state-behavior-response models, creating a comprehensive multi-level intervention system for dynamically adaptive precision interventions.

In this context, multicenter research designs further enhance the generalizability of findings. Considering that IGD clinical manifestations may be influenced by regional and cultural factors, conclusions from single-study samples often have limitations. Multiple studies [7, 8] have effectively improved the generalizability of prediction models by incorporating data from diverse geographical regions and socioeconomic populations. This research approach not only increases sample diversity but, more importantly, controls potential confounding factors, ensuring broader applicability of the conclusions.

Longitudinal data is crucial for multimodal prediction in understanding the development of IGD. Tracking changes in brain function and behavioral patterns over time through long-term studies provides valuable insights. Researchers can identify early indicators of addiction, enabling timely intervention before symptoms worsen. Analyzing patterns of brain function and behavior longitudinally has been shown to predict the likelihood of addiction, potentially preventing severe symptoms in vulnerable individuals [9]. Integrating multimodal data in longitudinal studies is essential for monitoring addiction's progression and enhancing early intervention strategies.

## CONCLUSION

In conclusion, IGD represents a pressing mental health concern characterized by a multifaceted nature influenced by various interconnected factors. Conventional single-method and cross-sectional research approaches prove inadequate in addressing the need for timely prediction and intervention. The integration of multimodal data, AI algorithms, and longitudinal data holds promise for enhancing the accurate identification of at-risk groups and the provision of personalized, evidence-based intervention strategies. This comprehensive methodology stands to effectively curb the proliferation of IGD and offer substantial support for adolescent mental well-being. Continued advancements in technology are anticipated to yield more precise and adaptable prediction and intervention protocols, thereby significantly reducing addiction vulnerabilities among adolescent populations.

## LIST OF ABBREVIATIONS

AI: Artificial Intelligence
IGD: Internet Gaming Disorder
rTMS: Repetitive Transcranial Magnetic Stimulation
tACS: Transcranial Alternating Current Stimulation
tDCS: Transcranial Direct Current Stimulation
